# The hidden nitrogen nexus: stochastic assembly and linear gene synergies drive urban park microbial networks

**DOI:** 10.3389/fmicb.2025.1652652

**Published:** 2025-10-01

**Authors:** Maoping Li, Jie Bi, Xiaochen Wang, Huan Li

**Affiliations:** ^1^State Key Laboratory of Herbage Improvement and Grassland Agro-Ecosystems, College of Pastoral Agriculture Science and Technology, Lanzhou University, Lanzhou, Gansu, China; ^2^Institute of Microbiome Frontiers and One Health, School of Public Health, Lanzhou University, Lanzhou, China

**Keywords:** nitrogen cycling genes, urban park environments, metagenomic binning, linear arrangements of genes, microbial networks

## Abstract

Urban parks play a significant role in environmental greening, cultural heritage, and recreational activities. The diversity and distribution of park environmental microbiota have become a hot focus of microbial ecology. However, there has been limited attention on the functional attributes of microbial communities, highlighting the importance of studying the distribution and diversity of functional genes in urban parks. Here, we employed metagenomic sequencing and binning to explore the diversity, assembly, and functional synergy of nitrogen cycling genes from the grassland soil and water in urban parks. Our results showed that glutamate metabolism and assimilatory nitrate reduction are the predominant nitrogen cycling pathways in both the soil and water. The diversity of nitrogen cycling genes in water was more abundant than in soil. The assembly of nitrogen cycling genes in both the soil and water was primarily driven by stochastic processes. Nutrient factors (such as total sulfur) were the most significant influencers of nitrogen cycling genes in park soil, while bacterial communities were the most critical determinants in water. The gene *narH*, involved in multiple nitrogen cycling metabolic pathways, was identified as an important marker of nitrogen storage in both soil and water. Through metagenomic binning, we discovered linear arrangements of multiple nitrogen cycling genes, such as *narG*-*narH*-*narJ*-*narI*, which collectively participate in the reduction of nitrate to nitrite, demonstrating the synergy, functional redundancy, and complementarity among nitrogen cycling genes. Our study holds significant implications for the biochemical cycling and the management of nitrogen pollution in urban parks.

## 1 Introduction

Terrestrial and aquatic environments are the most significant reservoirs of reactive nitrogen on Earth. On a global scale, biological nitrogen fixation and the use of nitrogen fertilizers are the primary sources of reactive nitrogen inputs (Pashaei et al., 2022). It is estimated that these processes contribute approximately 450 Tg of nitrogen to the atmosphere and biosphere annually (Fowler et al., 2015). On one hand, nitrogen is an essential element for living organisms, particularly as a fundamental component of proteins and nucleic acids (Berdalet et al., 1994). In plants, nitrogen is a constituent of chlorophyll, promoting their growth and development (Schlemmer et al., 2013). On the other hand, the nitrification-denitrification processes in soil produce nitrogen dioxide and nitrogen oxides, which are important greenhouse gases with significant impacts on global climate change (Barnard et al., 2005). Additionally, excessive nitrogen can lead to soil acidification, water eutrophication, and disruption of ecosystem balance (Groffman, 2008). Ammonia and nitrite can also be toxic to organisms (Romano and Zeng, 2013). The storage and transformation of nitrogen in the environment depend on microorganisms and associated nitrogen cycling genes. However, current research on nitrogen-cycling microorganisms and genes in the environment remains insufficient.

The nitrogen cycle refers to the process of mutual transformation between elemental nitrogen and nitrogen-containing compounds in ecosystems, including biological nitrogen fixation, ammonification, anaerobic ammonium oxidation, nitrification, denitrification, and dissimilatory nitrate reduction (Yang et al., 2023). Microorganisms can alter nitrogen-containing compounds through more than fourteen redox reactions. The enzymes that participate in these reactions are encoded through various nitrogen-cycling genes. For example, the conversion of atmospheric nitrogen to ammonia can be achieved by the nitrogen fixation gene *nifH* (Auman et al., 2001). *NirK* and *nirS* are rate-limiting enzyme genes in the denitrification process (Li et al., 2020), reducing nitrite to nitric oxide. *HzsA* and *hzsB* are used to detect anaerobic ammonium-oxidizing bacteria in the environment, converting ammonia to nitrogen gas (Han et al., 2017). Multiple genes and enzymes get involved in the conversion of different nitrogen valence states. Although some studies have explored nitrogen-cycling genes and related microorganisms in various natural ecosystems, such as ocean (Zehr and Kudela, 2011), soil (Wallenstein and Vilgalys, 2005), and forest (Levy-Booth et al., 2014), research on nitrogen cycling in urban ecosystems influenced by human activities is limited. Investigating nitrogen cycling processes in urban ecosystems is crucial for regulating urban ecological environments and nitrogen pollution.

Urban parks are a vital component of urban ecosystems, serving multiple roles in cities, including providing recreational spaces, cultural heritage preservation, and various ecological benefits (Sadeghian and Vardanyan, 2013). For instance, urban parks enhance green spaces, improve air quality and the urban environment, and plants absorb carbon dioxide while releasing oxygen, filtering atmospheric pollutants, and improving local microclimates (Ayub et al., 2021; Edeigba et al., 2024). They also help conserve water resources and protect biodiversity, playing a significant role in sustainable urban development (Konijnendijk et al., 2013; Kowarik et al., 2020). Urban ecosystems experience elevated nitrogen levels due to human activities such as industrial emissions, wastewater discharge, and fertilizer use, far exceeding those found in natural environments (Zhu et al., 2016). Owing to persistent pollutant accumulation, microbial activity and nitrogen transformation processes in urban park surface soils differ from those in natural ecosystems, demonstrating enhanced nitrification and denitrification rates (Zhu et al., 2004). Furthermore, urban water bodies are frequently contaminated with organic matter and nutrients, which accelerate nitrogen cycling and regulate microbial processes, consequently altering nitrogen speciation and distribution patterns (He et al., 2025). In urban ecosystems, diverse microbes possess specialized functional genes that drive various nitrogen conversion processes (Wang et al., 2018). These microbial nitrogen-cycling processes exhibit complex interplay, as demonstrated by recent studies: while *anammox* dominates nitrogen removal in urban rivers, high levels of *nirS* and *nosZ* genes show that denitrification also occurs (Yu et al., 2020). However, most existing functional studies predominantly rely on 16S rRNA gene-based metagenomic predictions, which inherently lack functional resolution for precise microbial process characterization. This methodological limitation has constrained our understanding of the complexity in urban nitrogen cycling networks. In-depth exploration of nitrogen cycling processes in urban park soils and water through metagenomics is essential for the rational management and regulation of urban nitrogen pollution, contributing to the healthy development of urban ecosystems.

To address the above issues, this study collected grassland soil and water samples in nine parks in Lanzhou City, in northwest China. Through 16S sequencing, metagenomic sequencing, and physicochemical analysis, we investigated nitrogen-cycling microorganisms and the functions of nitrogen-cycling genes. We assume that nitrogen cycling gene pathways are different in soil and water. This study aims to answer the following questions: (1) whether there are differences in nitrogen-cycling microorganisms between park grassland soils and water? (2) What are the differences in nitrogen-cycling genes and pathways between park grassland soils and water? (3) Which factors influence the assembly of nitrogen-cycling genes in park grassland soils and water? (4) Do nitrogen-cycling genes in microorganisms exhibit synergy, redundancy, and functional complementarity?

## 2 Materials and methods

### 2.1 Sampling and physicochemical analysis

Soil and water samples were collected from nine urban parks in Lanzhou City, Gansu Province, China (36 °03′38′′ N, 103 °49′36′′ E) between April and June 2022. The parks included Jincheng Park (J), Yintan Wetland Park (Y), Longyuan Park (L), Jincheng Bonsai Garden (P), Xiaoxihu Park (X), Lanzhou Waterwheel Park (S), Yantan Park (T), Lanzhou Civic Square (G), and Xinyue Lake (H). At each of the nine sampling sites, two distinct types of samples were collected in a spatially paired design: a water sample and an adjacent grassland soil sample. Three sampling points were chosen in each park, resulting in a total of 27 grassland soil samples and 27 water samples. We collected 6 L water (5.5 L for microbial filtration and 0.5 L for physicochemical analysis) and adjacent 50 g grassland soil (using five-point sampling) for each sample. Detailed sampling site information and methods are described in our previous study (Wang et al., 2024). The collected samples were analyzed for a comprehensive suite of physicochemical parameters, including pH, oxidation-reduction potential (ORP), conductivity (CON), total dissolved solids (TDS), salinity, total carbon (TC), total nitrogen (TN), total phosphorus (TP), and total sulfur (TS), as well as heavy metals [mercury (Hg), arsenic (As), and lead (Pb)]; dissolved oxygen (DO) was specifically measured for water samples. The detailed methodologies and data available are described in the previous study (Wang et al., 2024).

### 2.2 Metagenomic sequencing, 16S rRNA gene sequencing, and analysis

Details of DNA extraction, metagenomics sequencing and bioinformatics analysis can be found in our previous study (Wang et al., 2024). Briefly, libraries were prepared using the TruSeq Nano DNA LT Kit (FC121-4001). Paired-end 2 × 150 bp sequencing was performed on the Illumina HiSeq 4000 platform at LC Sciences (Hangzhou, China). Single-gene abundance was assessed using transcripts per million (TPM) (Wagner et al., 2012). PCR amplicons for 16S rRNA gene sequencing were sequenced (2 × 250 bp paired-end) on the Illumina MiSeq system (Illumina, San Diego, CA, USA). The remaining sequences were clustered into operational taxonomic units (OTUs) at 97% similarity using the QIIME (Quantitative Insights Into Microbial Ecology) platform (Zahn, 2022). The longest reads were selected as representative sequences, and taxonomic information was annotated based on the Ribosomal Database Project (RDP) (Cole et al., 2003). OTUs belonging to mitochondria, chloroplasts, and non-bacterial sequences were removed. The OTU table was normalized using the “Daisyhopper” script (Gilbert et al., 2009), resulting in 4721 normalized sequences per sample. Raw sequence data from metagenomic and 16S rRNA gene sequencing were uploaded to the European Nucleotide Archive under accession numbers PRJEB74256 (metagenomic data) and PRJEB71977 (16S data).

### 2.3 Nitrogen cycle functional gene annotation and analysis

Non-redundant gene sequences were aligned with the NCycDB database (accessed December 2023)^1^ using DIAMOND v0.7.12 (Buchfink et al., 2015) to obtain functional annotations (Tu et al., 2019). Target sequences were extracted using the Seqtk program^2^, and taxonomic profiles of nitrogen-cycling communities were obtained (Shen et al., 2016). Corresponding functional pathways were identified in the Kyoto Encyclopedia of Genes and Genomes (KEGG) database (release_84.0)^3^ (Kanehisa et al., 2017). Additionally, alpha diversity (Simpson and Shannon diversity) and beta diversity (based on Bray-Curtis distance matrices) of nitrogen-cycling genes and bacterial communities were calculated using the QIIME platform. Bray-Curtis community dissimilarity was also calculated based on Bray-Curtis distance matrices.

### 2.4 Metagenomic binning analysis

Paired-end sequences from all samples were merged, and contig sequences were assembled using Megahit (Li et al., 2015). Contigs were binned using MaxBin2, metaBAT2, and CONCOCT (Wu and Singer, 2021) to obtain metagenome-assembled genomes (MAGs). Bins with completeness >50% and contamination <10% were selected for further analysis using the CheckM database (Parks et al., 2015). The average abundance of bins was quantified using Salmon (Patro et al., 2017). Taxonomic annotation of contigs was performed using Taxator-tk based on the NCBI_nt and NCBI_tax databases (Dröge et al., 2015). Gene annotation was performed using PROKKA to obtain gene sequences and corresponding annotation information for each bin (Seemann, 2014).

### 2.5 Bioinformatics and statistical analysis

Differences in the abundance of nitrogen-cycling genes and microorganisms between grassland soil and water were calculated using one-way ANOVA and the Mann-Whitney U test (Yang et al., 2023). Linear discriminant analysis (LEfSe) was used to identify species with significant differences between grassland soil and water. Linear regression analysis (LDA) was used to estimate the effect size of each species’ abundance on the differences, and the R package “microeco” was used to display the evolutionary relationships of differentially abundant species (Liu et al., 2021). Differences in alpha diversity (Shannon and Simpson indices) between groups were calculated using the *t*-test. Beta diversity was visualized using principal coordinate analysis (PCoA) based on Bray-Curtis and Jaccard distance matrices, and significance was calculated using permutational multivariate analysis of variance (PERMANOVA) (Rothenberg et al., 2022). Differences in nitrogen-cycling genes and microbial communities between grassland soil and water were compared using the Mann-Whitney U test.

To comprehensively explore the drivers of nitrogen cycling gene abundance and pathway potential, we performed Spearman correlation analysis. The analyzed variables included two categories: (1) Physicochemical Factors: a suite of measured environmental parameters (e.g., pH, temperature, NH_4_^+^-N, NO_3_^–^-N, TOC, etc.). (2) Microbial Community Metrics: to capture different aspects of microbial community ecology, we included alpha and beta diversity. Alpha diversity represented by the Shannon index (H’), which was calculated to assess the species diversity (richness and evenness) within each individual sample. Beta diversity represented by the sample scores on the first two principal coordinates (PCoA1 and PCoA2) derived from a Principal Coordinates Analysis (PCoA). The PCoA was performed on a Bray-Curtis dissimilarity matrix, which was generated from the microbial community composition data. PCoA1 and PCoA2 thus serve as synthetic, continuous variables that summarize the major gradients in overall microbial community structure (i.e., compositional dissimilarity) across all samples. By including these microbial community metrics alongside physicochemical factors in the correlation analysis, we aimed to evaluate and compare the relative influence of the abiotic environment, local community diversity, and overall community composition on nitrogen cycling processes.

Spearman correlations between nitrogen-cycling genes were calculated using the R package “psych”, retaining correlations with |r| > 0.4 and *P* < 0.05. Co-occurrence networks of nitrogen-cycling genes were visualized using Gephi v0.10.1 (Bastian et al., 2009), and network topological parameters (including nodes, edges, average degree, density, diameter, modularity, average clustering coefficient, and average path length) were calculated. The community assembly process of nitrogen-cycling genes was calculated based on Bray-Curtis distance matrices and a null model (With, 1997), and the proportion of stochastic processes (%) was determined. The neutral community model (NCM) was used to further quantify the importance of stochastic processes in the assembly of nitrogen-cycling gene communities (Sloan et al., 2006). Spearman correlations between nitrogen-cycling genes and environmental physicochemical factors and bacterial community diversity were calculated, and heatmaps were generated using the R packages “pheatmap” and “ComplexHeatmap” (Gu and Hübschmann, 2022). The importance of these variables was further assessed using the random forest model in the R package “randomForest” (Breiman, 2001). The partial least squares path model (PLS-PM) in the R package “plspm” was used to analyze the direct and indirect effects of influencing factors on nitrogen-cycling genes (Hulland, 1999). Gene clusters were visualized using the Gene Structure Display Server (GSDS2.0) (Hu et al., 2015).

## 3 Results

### 3.1 Differences in nitrogen cycle genes and pathways in park grassland soil and water

**FIGURE 1:**
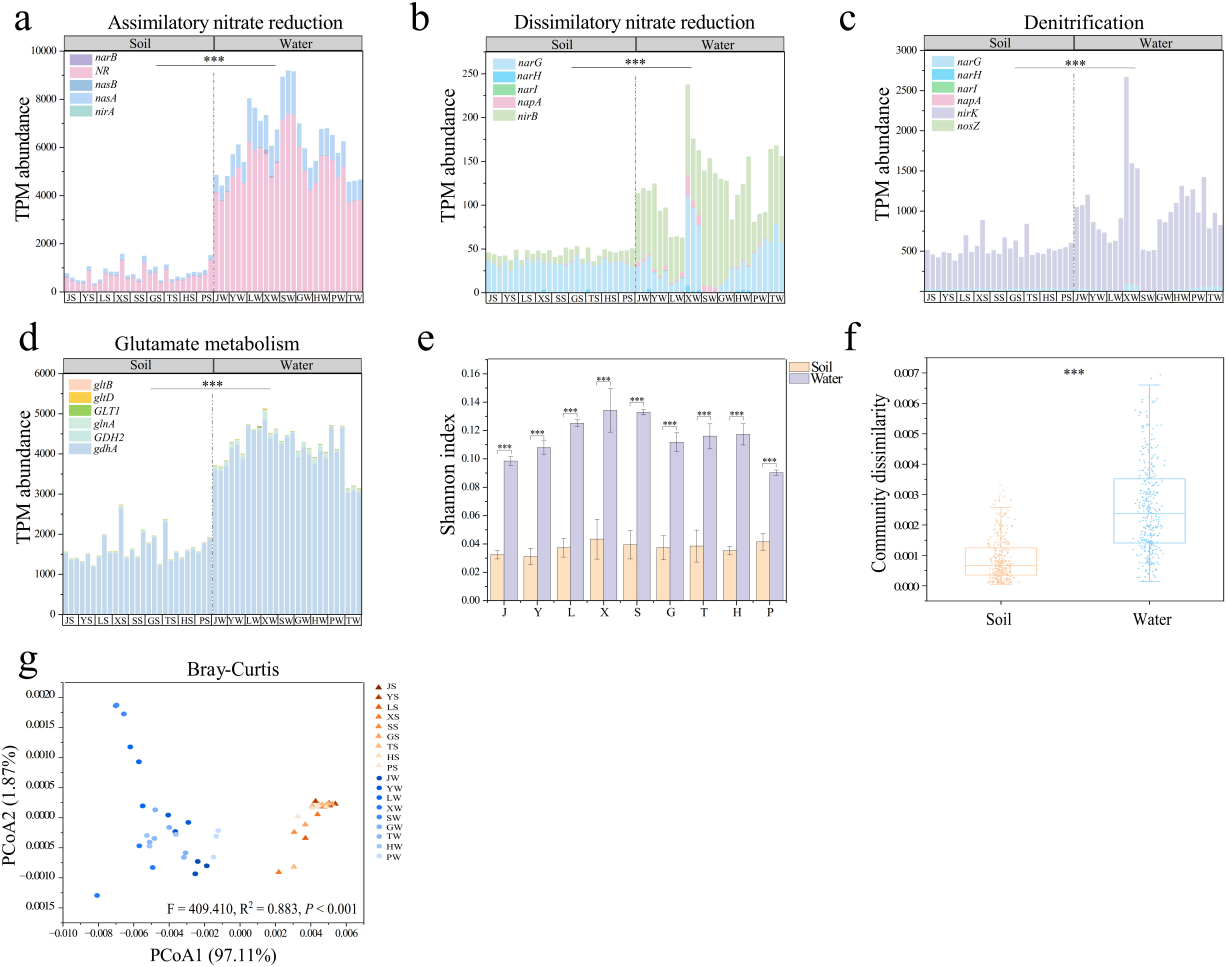
Abundance and diversity of nitrogen cycling genes in park grassland soils and water. TPM abundance of nitrogen cycling genes in each pathway **(a–d).** The alpha diversity of nitrogen cycling genes **(e)**. Intra-community dissimilarity based on the Bray-Curtis distance matrix **(f)** and PCoA **(g)** demonstrated beta diversity of nitrogen cycle genes. In the PCoA plot, PERMANOVA was used to test for variability in community structure among soil and water groups in park meadows. Mann-Whitney U test was used to test the significance of differences in nitrogen cycling gene abundance and community structure in park grassland soils and water. ***P < 0.001. PCoA, principal coordinate analysis; J, Jincheng Park; Y, Yintan Wetland Park; L, Longyuan Park; X, Xiaoxihu Park; S, Lanzhou Waterwheel Park; G, Lanzhou Civil Square; P, Jincheng Bonsai Park; T, Yantan Park; H, Xinyue Lake. “S” meant the soil samples, and “W” meant the water samples.

We identified a total of 24 nitrogen cycle genes in all samples from park grassland soil and water (Supplementary Figure 1). These genes were categorized into six functional modules based on their pathways: Assimilatory nitrate reduction to ammonium (ANRA), Dissimilatory nitrate reduction to ammonium (DNRA), Denitrification, and Glutamate metabolism. No genes related to Nitrogen fixation were detected. The primary nitrogen cycle pathways in park grassland soil were Glutamate metabolism (average TPM abundance of 1660.650), ANRA (average TPM abundance of 820.769), and Denitrification (average TPM abundance of 537.742) (Figures 1a–d). In water, the main pathways were Glutamate metabolism (average TPM abundance of 8396.159), and ANRA (average TPM abundance of 1267.056) (Figures 1a–d). The nitrogen cycle genes with the highest TPM abundance in soil were *gdhA* (average TPM abundance of 1613.240), *narB* (average TPM abundance of 657.983), and *nirK* (average TPM abundance of 502.428), while in water, they were *narB* (average TPM abundance of 5196.593), *gdhA* (average TPM abundance of 4079.150), and *nasA* (average TPM abundance of 1107.007) (Supplementary Figure 3). Additionally, the TPM abundance of nitrogen cycle genes involved in ANRA, DNRA, Denitrification, and Glutamate metabolism was significantly higher in park water than in grassland soil (Mann-Whitney U test, *P* < 0.001).

The alpha diversity (Shannon and Simpson indices) of nitrogen cycle genes in park water was significantly higher than in grassland soil (Figure 1e and Supplementary Figure 2b, *T*-test, *P* < 0.001). PCoA analysis based on Bray-Curtis and Jaccard distance matrices revealed significant differences in the community structure of nitrogen cycle genes between park grassland soil and water (Figure 1g, Supplementary Figure 2c and Supplementary Table 1, PERMANOVA, *P* < 0.001). Further analysis showed that the variability within nitrogen cycle gene communities was significantly higher in water than in soil (Figure 1f, Supplementary Figure 2d, Mann-Whitney U test, *P* < 0.01).

**FIGURE 2:**
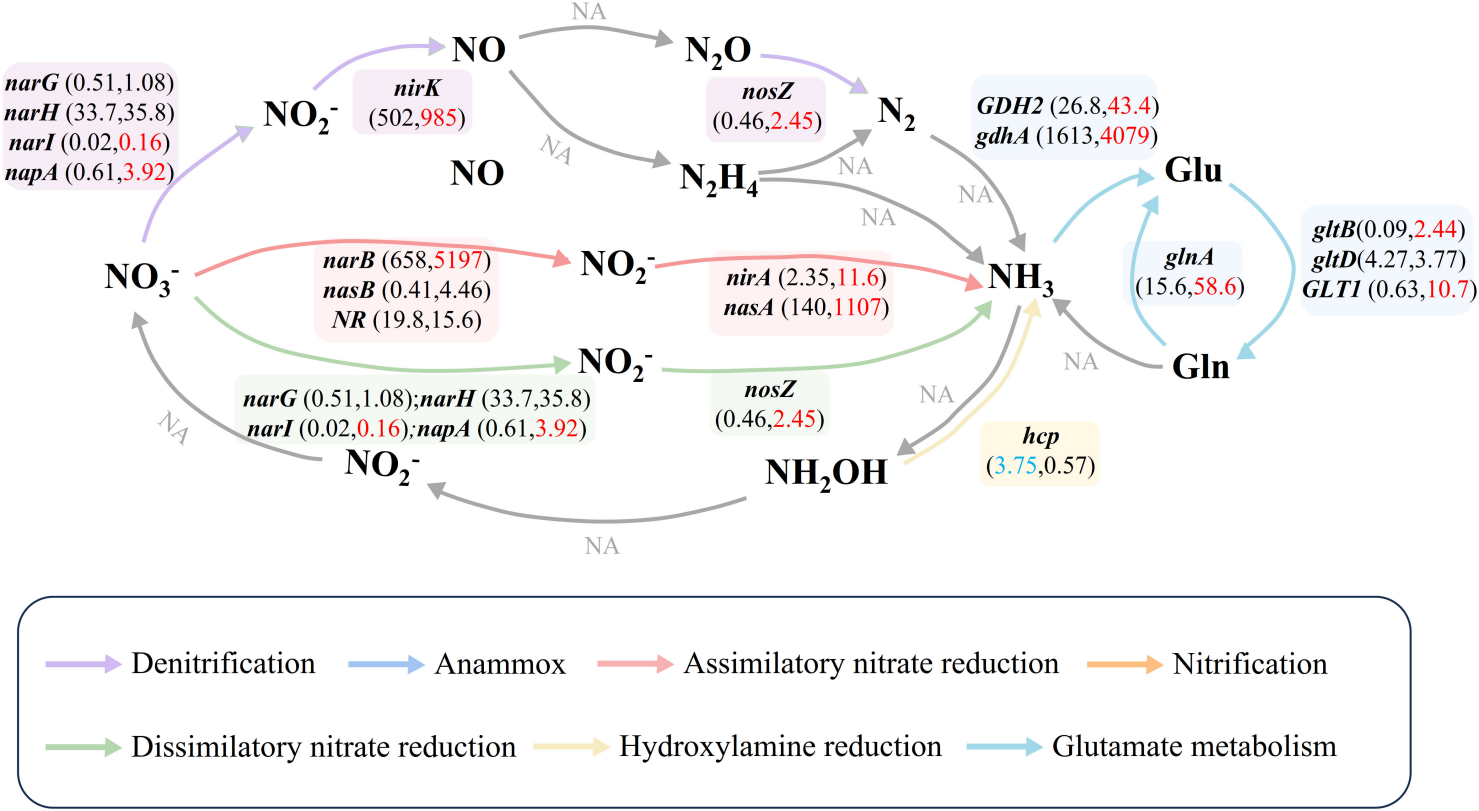
Nitrogen cycle pathways and nitrogen cycle gene abundance. Different colored arrows represented different nitrogen transformation pathways. Gray arrows and NA indicated that nitrogen cycle genes involved in the pathway were not detected in the samples. Numbers in parentheses represent the average abundance of the gene in park grassland soil (former) and water (latter). Red numbers indicate that the gene is enriched in water, while blue indicates that the gene is enriched in grassland soils. One-Way ANOVA was used to test for differences in nitrogen cycling gene abundance in park grassy soils and water.

The heatmap-enrichment bar chart (Supplementary Figure 3) displayed nitrogen cycle genes significantly enriched in grassland soil and water. Ten nitrogen cycle genes were significantly enriched in park water (Supplementary Table 2, One-way ANOVA, *P* < 0.05), with *napA* showing the highest enrichment fold (26.66). In contrast, *hcp* and *nrfC* were significantly enriched in grassland soil. Based on the KEGG PATHWAY database, we constructed nitrogen cycle pathways (Figure 2), revealing that the primary nitrogen cycle pathway in park grassland soil was Hydroxylamine reduction, while in water, the main pathways were ANRA, DNRA, and Glutamate metabolism.

### 3.2 Differences in microorganisms carrying nitrogen cycle genes in park grassland soil and water

**FIGURE 3:**
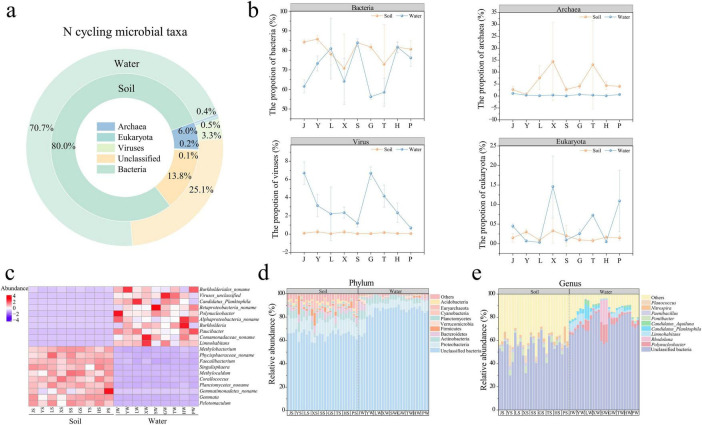
Composition of nitrogen-cycling microbial taxa in park grassland soils and water **(a).** Proportion of bacteria, fungi, viruses and eukaryotes in each park grassland soil and water **(b).** Heat map **(c)** showing the top 10 microbial genera significantly enriched in park grassland soil and water, respectively (One-way ANOVA, *P* < 0.05). Composition of nitrogen-cycling microorganisms at the phylum level **(d)** and genus level **(e)**. PCoA, oprincipal coordinate analysis; J, Jincheng Park; Y, Yintan Wetland Park; L, Longyuan Park; X, Xiaoxihu Park; S, Lanzhou Waterwheel Park; G, Lanzhou Civil Square; P, Jincheng Bonsai Park; T, Yantan Park; H, Xinyue Lake. “S” meant the soil samples, and “W” meant the water samples.

Nitrogen cycle microorganisms (Figure 3a) were primarily composed of bacteria (soil: 80.0%; water: 70.7%), archaea (soil: 6.0%; water: 0.4%), viruses (soil: 0.1%; water: 3.3%), and eukaryotes (soil: 0.2%; water: 0.5%). Bacteria (Mann-Whitney U test, *P* < 0.01) and archaea (Mann-Whitney U test, *P* < 0.001) were significantly more abundant in grassland soil, indicating their key roles in soil-specific nitrogen processes, while viruses (Mann-Whitney U test, *P* < 0.001) were more prevalent in water, potentially influencing microbial community dynamics and nutrient cycling through lysis. Eukaryotes showed no significant difference between soil and water (Mann-Whitney U test, *P* > 0.05) (Figure 3b). At the phylum level (Figure 3d), the dominant nitrogen cycle microorganisms in park grassland soil and water were unclassified bacteria (64.41%, 84.53%), *Proteobacteria* (11.32%, 7.24%), *Actinobacteria* (5.11%, 4.08%), and *Bacteroidetes* (4.37%, 1.55%). The high proportion of unclassified bacteria in both habitats highlights a vast, unexplored reservoir of nitrogen-cycling potential. At the genus level (Figure 3e), the main nitrogen cycle microorganisms in grassland soil were unclassified bacteria (52.23%, 73.16%), Pontibacter (2.77%), Planococcus (1.75%), and Nitrospira (1.48%), the latter being a known nitrite-oxidizing genus crucial for nitrification, while in water, they were Polynucleobacter (4.83%), Rhodoluna (3.10%), and Limnohabitans (2.97%), genera often associated with the degradation of organic nitrogen.

The alpha diversity (Shannon and Simpson indices) of nitrogen cycle microorganisms in grassland soil was significantly higher than in water (Supplementary Figures 4a, b, T-test, *P* < 0.05), suggesting a greater functional redundancy in soil nitrogen cycling. The community composition of nitrogen cycle microorganisms differed significantly between park grassland soil and water (Supplementary Table 3, PERMANOVA, *P* < 0.001), with soil communities being more densely distributed and water communities more dispersed (Supplementary Figures 4c, d). This structural divergence points to fundamentally different nitrogen cycling strategies in the two ecosystems. Further analysis showed that the variability within nitrogen cycle microbial communities was significantly higher in water than in soil (Supplementary Figures 4e, f, Mann-Whitney U test, *P* < 0.001), implying potentially more dynamic nitrogen cycling processes in the aquatic environment.

One-way ANOVA (Supplementary Table 4) identified nitrogen cycle microorganisms with significantly different abundances in grassland soil and water. The heatmap (Figure 3c) shows the top 10 enriched microbial genera in soil and water. In park grassland soil, the top three enriched nitrogen cycle microorganisms were *Planctomycetes*_noname, *Faecalibacterium*, and Phycisphaeraceae_noname, which may contribute to anaerobic nitrogen processes and organic matter turnover, while in water, they were *Limnohabitans*, *Polynucleobacter*, and *Paucibacter*. Lefse analysis (Supplementary Figure 5) also revealed that enriched nitrogen cycle microorganisms in grassland soil were primarily bacteria and archaea, with *Proteobacteria*, *Bacteroidetes*, and *Firmicutes* being the dominant phyla, together underpinning a broad range of soil nitrogen transformations. In water, enriched microorganisms were mainly unclassified bacteria and viruses.

**FIGURE 4:**
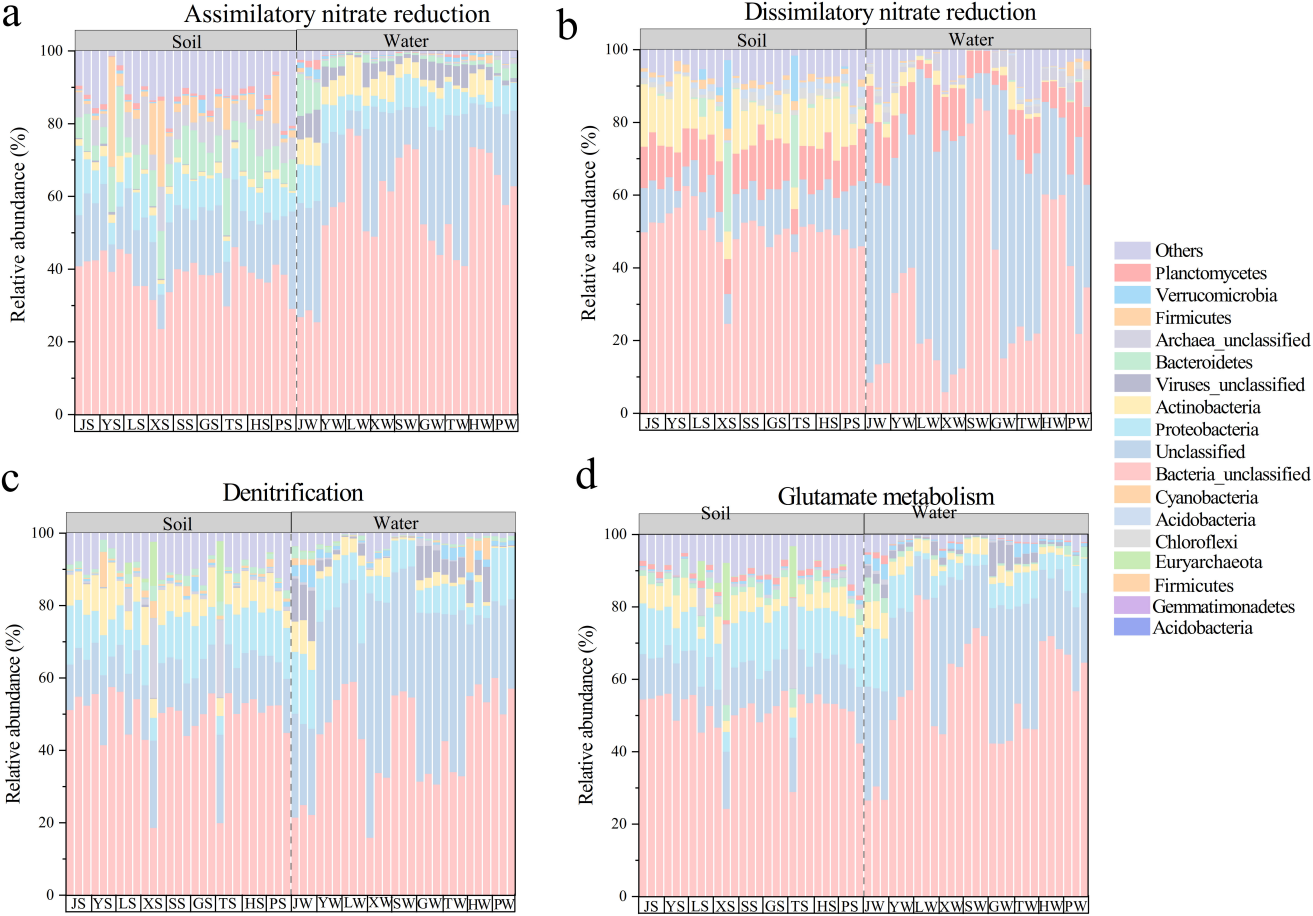
Composition of the top 10 nitrogen cycling microorganisms at the genus level of abundance for four different pathways: **(a)** Assimilatory nitrate reduce. **(b)** Dissimilatory nitrate reduce. **(c)** Denitrification. **(d)** Glutamate metabolism. J, Jincheng Park; Y, Yintan Wetland Park; L, Longyuan Park; X, Xiaoxihu Park; S, Lanzhou Waterwheel Park; G, Lanzhou Civil Square; P, Jincheng Bonsai Park; T, Yantan Park; H, Xinyue Lake. “S” meant the soil samples, and “W” meant the water samples.

At phylum-level, microorganisms involved in different nitrogen cycle pathways were predominantly unclassified bacteria and unclassified microorganisms, underscoring that the core agents of nitrogen cycling remain largely uncharacterized (Figure 4). Additionally, the composition of nitrogen cycle microorganisms varied across pathways, with differences between soil and water. For example, *Bacteroidetes* were the main microorganisms involved in ANRA in soil, while Proteobacteria dominated in water. *Planctomycetes* were the main microorganisms involved in DNRA, and *Proteobacteria* were the primary microorganisms involved in Denitrification, and Glutamate metabolism. This clear functional differentiation suggests that soil tends to conserve nitrogen through processes like DNRA, whereas water systems are more oriented toward nitrogen loss via denitrification.

### 3.3 Differences in co-occurrence networks of nitrogen cycle genes in park grassland soil and water

**FIGURE 5:**
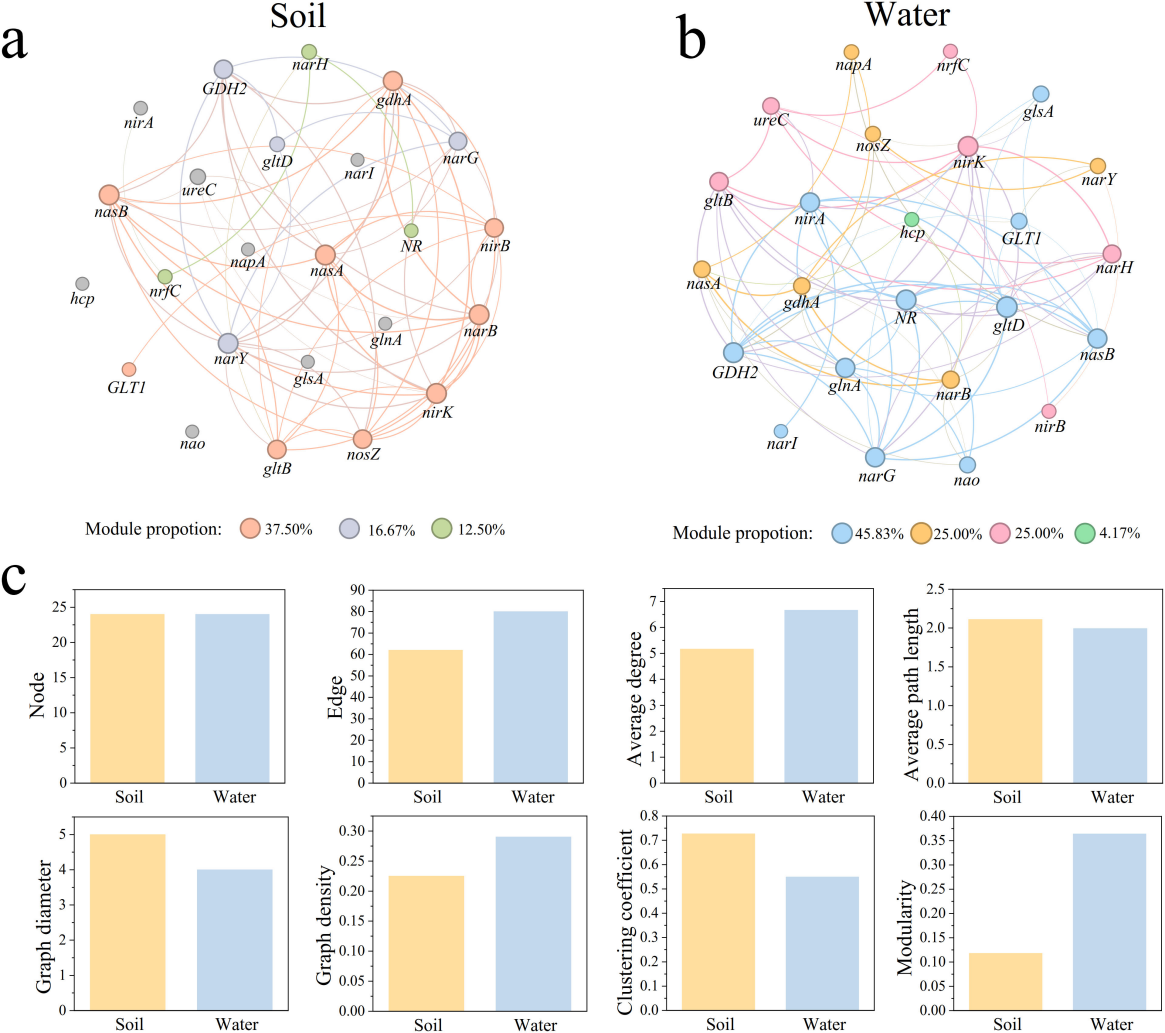
Co-occurring networks of nitrogen cycling genes in park grassland soil **(a)** and water **(b)** and network topology characterization **(c).** Nodes in the network indicate nitrogen cycle genes. A connecting line between nodes indicates a significant correlation between two nitrogen cycle genes (Spearman correlation, |r| > 0.4, *P* < 0.05). Nodes and edges are colored according to modularity.

We constructed co-occurrence networks of nitrogen cycle genes with significant correlations in park grassland soil (Figure 5a) and water (Figure 5b) (|r| > 0.4, *P* < 0.05). Analysis of the topological characteristics of the two networks (Figure 5c and Supplementary Table 5) revealed that the number of edges, average degree, and graph density were lower in the soil network than in the water network, indicating greater complexity in the water network. The average path length and graph diameter were higher in the soil network, suggesting tighter connections between nitrogen cycle genes in water. Additionally, the modularity of the water network was higher. Both soil and water networks were dominated by positive edges (soil: 90.323%, water: 72.500%). In the soil network, *narB* had the most associations with other genes, showing significant positive correlations with *nasA*, *gdhA*, *nirK*, *nasB*, and *GDH2*, and a significant negative correlation with *ureC*. In the water network, *gltD* had the most associations, showing significant positive correlations with *narG*, *nasB*, *gltB*, and *narH*, and significant negative correlations with *narB*, *glsA*, and *GLT1*.

### 3.4 Community assembly mechanisms of nitrogen cycle genes in park grassland soil and water

**FIGURE 6:**
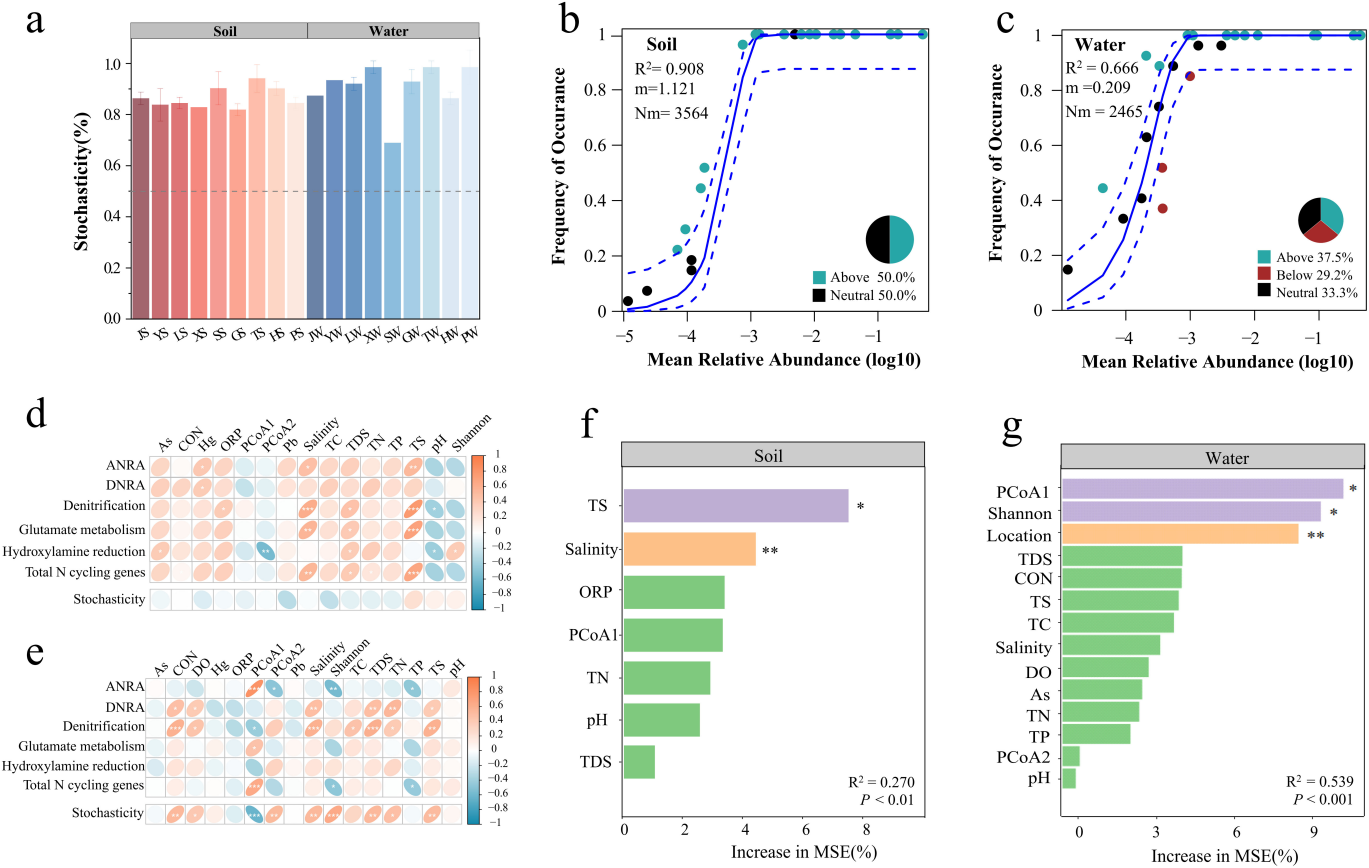
Mechanisms and influencing factors of community assembly of nitrogen cycling genes in park grassland soil and water. The percentage of stochasticity in the process of nitrogen cycle gene community assembly was calculated by a null model **(a)**. Neutral community models (NCM) were fitted to fit the contribution of stochastic processes in the assembly of nitrogen-cycling gene communities **(b,c).** R^2^ denotes how well the neutral model matches the actual data, m denotes the migration rate of the community, and Nm denotes the amount of migratory diffusion. The blue dashed line represents the 95% confidence interval of the model (estimate obtained by 1000 bootstrap), and genes occurring at frequencies above, in line with, and below the NCM prediction are shown in green, black, and red, respectively. Spearman correlations of physicochemical factors with nitrogen cycling pathways, total nitrogen cycling gene abundance, and community stochastic processes in park grassland soils and water **(d,e)**. Random Forest Modeling to Assess Important Environmental Variables Driving Nitrogen Cycling Genes in Park Meadow Soil **(f)** and Water **(g)**. R^2^ denoted the overall fit of the model, *P*-value denoted the significance of the full model, * denoted the significance of the variable, *P < 0.05, **P < 0.01. J, Jincheng Park; Y, Yintan Wetland Park; L, Longyuan Park; X, Xiaoxihu Park; S, Lanzhou Waterwheel Park; G, Lanzhou Civil Square; P, Jincheng Bonsai Park; T, Yantan Park; H, Xinyue Lake. “S” meant the soil samples, and “W” meant the water samples. ANRA, assimilatory nitrate reduction to ammonium; DNRA, dissimilatory nitrate reduction to ammonium; PCoA, principal coordinate analysis; CON, conductivity; ORP, oxidation-reduction potential; TDS, total dissolved solids; DO, dissolved oxygen; TC, total carbon; TN, total nitrogen; TP, total phosphorus; TS, total sulfur; Hg, mercury; As, arsenic; Pb, lead; Shannon, the alpha diversity of the bacterial community; PCOA1, PCOA2, the beta diversity of the bacterial community; Increase in MSE(%), percentage of increase of mean square error.

The null model results indicated that the assembly of nitrogen cycle gene communities in park grassland soil and water was primarily driven by stochastic processes (Figure 6a), with no significant difference in the proportion of stochasticity between soil and water (One-way ANOVA, *P* > 0.05). Further analysis using the neutral community model (NCM) to assess the importance of stochastic processes (Figures 6b, c) showed that the NCM R^2^ was higher for soil than for water, indicating a better overall fit and a closer approximation to the neutral model. This suggests that the assembly of nitrogen cycle gene communities in grassland soil was more influenced by stochastic processes and less by deterministic processes. The migration rate (*m* value) of nitrogen cycle genes was higher in grassland soil, indicating lower diffusion limitation, while the nitrogen cycle gene communities in water were more susceptible to diffusion limitation.

### 3.5 Drivers of nitrogen cycle genes

The correlation heatmap of environmental physicochemical factors and nitrogen cycle genes showed that different genes within the same pathway did not necessarily respond similarly to environmental factors (Supplementary Figure 6). For example, salinity showed a significant negative correlation with *narG*, *narH*, *narY*, and *nirK* involved in Denitrification in water, but no significant correlation with *napA* and *nosZ*. Arsenic content in soil showed a significant negative correlation with *nasA* but no significant correlation with other genes in the ANRA pathway, such as *narB*, *NR*, *nirA*, and *nasB*. TS showed a significant positive correlation with *nirA* in soil but a significant negative correlation with *nasA* and *nasB*. The alpha diversity of bacterial communities showed a significant positive correlation with *gdhA* involved in Glutamate metabolism in water but a significant negative correlation with *glnA*.

Spearman correlation analysis of physicochemical factors with nitrogen cycle pathways, total nitrogen cycle gene abundance, and community assembly in park grassland soil and water showed that salinity, TDS, and TS were significantly positively correlated with total nitrogen cycle gene abundance in soil (Figure 6d), while the beta diversity of bacterial communities was significantly positively correlated with total nitrogen cycle gene abundance in water (Figure 6e). In grassland soil, physicochemical factors showed no significant correlation with the assembly of nitrogen cycle gene communities, while in water, CON, DO, bacterial community diversity, salinity, TC, TDS, TN, TP, and TS were significantly correlated with the assembly of nitrogen cycle gene communities. TN showed a significant positive correlation with DNRA, and Denitrification in water. Further analysis of the relationship between TN and nitrogen cycle genes (Supplementary Figure 7) revealed that *narH* and *narB* were significantly positively correlated with TN in soil (Spearman, *P* < 0.05), while other nitrogen cycle genes showed no significant correlation. In water, *narH*, *nirA*, *nirK*, *GDH2*, *glnA*, *hcp*, *ureC*, and *gltB* were significantly positively correlated with TN (Spearman, *P* < 0.05). Random forest models identified TS and salinity as key predictors of nitrogen cycle genes in park grassland soil (Figure 6f), while bacterial community diversity and geographic location were key predictors in water (Figure 6g).

**FIGURE 7:**
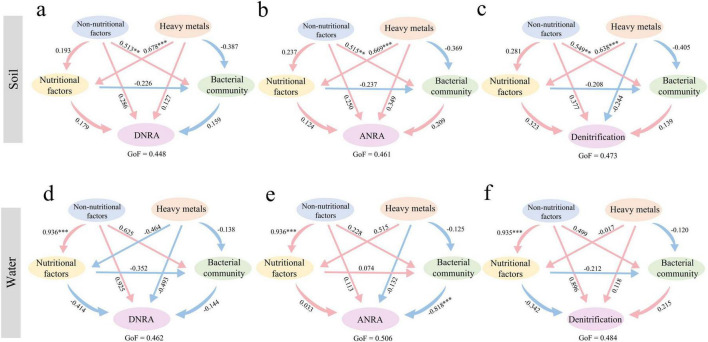
Potential mechanisms of influence of individual nitrogen cycling gene pathways in park grassland soils [**(a)** DNRA, **(b)** ANRA, **(c)** Denitrification] and water [**(d)** DNRA, **(e)** ANRA, **(f)** Denitrification] based on PLS-PM. The pink and blue arrows in the figure indicate positive and negative impacts, respectively, and the numbers next to the arrows represent normalized path coefficients. *P < 0.05, **P < 0.01, ***P < 0.001. GoF, goodness of fit.

To explore the potential mechanisms by which heavy metals, non-nutrient physicochemical factors, nutrient factors, and bacterial community diversity influence nitrogen cycle genes in grassland soil and water, we used partial least squares path modeling (PLS-PM) and standardized effect analysis. The results showed that non-nutrient physicochemical factors were the primary drivers of nitrogen cycle genes in park grassland soil, followed by heavy metals and nutrient factors, all of which had direct positive effects (Supplementary Figures 8a, c). Bacterial community diversity had a significant direct negative effect on nitrogen cycle genes in water and was the primary driver, followed by the positive effect of nutrient factors (Supplementary Figures 8b, d). Further analysis of the influence of these factors on specific nitrogen cycle pathways (Figure 7) revealed that environmental physicochemical factors were the primary drivers of Denitrification in both grassland soil and water. The primary drivers of ANRA and DNRA differed between soil and water. Non-nutrient physicochemical factors had a direct positive effect on ANRA in soil, while bacterial community diversity had a significant direct negative effect on ANRA in water. Heavy metals were the primary drivers of DNRA in soil, while non-nutrient physicochemical factors were the primary drivers in water.

### 3.6 Metagenomic binning analysis

**FIGURE 8:**
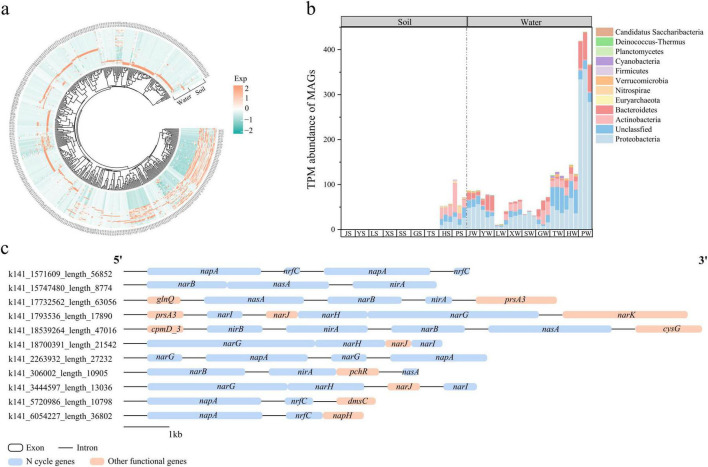
Relative abundance of MAGs assembled to nitrogen cycling genes in park grass soil and water samples **(a).** Composition of MAGs containing nitrogen cycling genes at the gate level **(b).** Partial clustering of genes containing nitrogen cycling genes **(c).** Orange and blue regions represent coding sequences and black lines represent introns. MAGs, metagenome-assembled genomes; J, Jincheng Park; Y, Yintan Wetland Park; L, Longyuan Park; X, Xiaoxihu Park; S, Lanzhou Waterwheel Park; G, Lanzhou Civil Square; P, Jincheng Bonsai Park; T, Yantan Park; H, Xinyue Lake. “S” meant the soil samples, and “W” meant the water samples.

A total of 379 MAGs containing nitrogen cycle genes were assembled from park grassland soil and water samples. Of these, 281 MAGs were assembled from grassland soil, and 285 MAGs were assembled from water (Figure 8a). The dominant phyla of MAGs containing nitrogen cycle genes in grassland soil were *Actinobacteria*, *Proteobacteria*, and *Euryarchaeota*, while in water, they were *Proteobacteria*, *Bacteroidetes*, and *Actinobacteria* (Figure 8b). Additionally, we identified some fragments carrying nitrogen cycle genes in these MAGs (Figure 8c). In bins containing nitrogen cycle genes, *narG*-*narH*-*narJ*-*narI* formed a common linear structure responsible for the reduction of nitrate to nitrite. The gene *napA*, which also participates in this reduction, often co-occurred with *narG* or *nrfC* (a gene involved in energy metabolism). Furthermore, *narB*-*nasA*-*nirA* collectively participated in the ANRA pathway. These results provide evidence of the synergistic interactions among nitrogen cycle genes, revealing their redundancy, complementarity, and functional coordination.

## 4 Discussion

### 4.1 Microorganisms carrying nitrogen cycle genes in the park environment exhibit diverse characteristics

The nitrogen cycle microorganisms in the park are primarily bacteria, accounting for 70%–80% of the total, followed by archaea, viruses, and eukaryotes. This result is similar to previous studies on nitrogen cycle microorganisms in polluted water environments (Yang et al., 2023), both confirming that bacteria are the main force in nitrogen cycle microorganisms. Sixty percent of the nitrogen cycle groups are identified as unclassified bacteria, indicating that many nitrogen cycle microorganisms in the park environment may be unidentified new bacterial groups. The alpha diversity of soil nitrogen cycle microorganisms is significantly higher than that of water environments, probably due to the higher microbial richness in soil environments, which can accommodate more nitrogen cycle microorganisms. Compared to the park soil environment, the park water environment is enriched with *Limnohabitans*, which are widely distributed in freshwater ecosystems and have strong environmental adaptability, rapid growth and reproduction capabilities, and substrate uptake capabilities (Props and Denef, 2020). These characteristics are of significant ecological importance for nitrogen transformation in water environments. In contrast, the park soil is more enriched with *Planctomycetes*, which have been identified as a rich nitrogen-fixing microbial group in ocean microbial studies (Delmont et al., 2018).

We found that different microorganisms participate in different nitrogen cycle processes, indicating a clear division of labor among microorganisms in the nitrogen cycle. Consistent with the highly diverse lineages of global nitrogen cycle microorganisms (Nelson et al., 2016), this suggests that diverse microorganisms maintain the nitrogen cycle process, possibly due to functional redundancy (Philippot et al., 2013). For example, the main microorganisms involved in the ANRA pathway in park grassland soil are *Bacteroidetes*, while those involved in the nitrification pathway are mainly *Actinobacteria*. Additionally, microorganisms involved in the same nitrogen cycle process in soil and water differ. For instance, the main microorganisms involved in the nitrification pathway in grassland soil are *Actinobacteria*, while in water, they are *Proteobacteria*. This may be due to different environmental conditions in soil and water. For example, the park water environment has higher carbon, nitrogen and sulfur content, while the soil has higher phosphorus content. It has shown that soil microbial communities were mainly regulated by carobon and nitrogen rather than phosphorus and sulfur (Tang et al., 2023). While nitrogen and phosphorus were the main determining factors for aquatic microbiota (Sun et al., 2021). These differences in nutrient elements filter and select for different microbial enrichments, and thereby lead to the difference of nitrogen-cycle microbes.

### 4.2 There are different nitrogen metabolism in park soil and water

In this study, we detected 24 nitrogen cycle genes in park soil and water environments, including functional modules such as Assimilatory nitrate reduction to ammonium (ANRA), Dissimilatory nitrate reduction to ammonium (DNRA), Glutamate metabolism, Denitrification. Consistent with previous studies (Song et al., 2022), we found that glutamate metabolism and nitrate reduction are the main nitrogen cycle processes in park soil and water environments, as organic nitrogen metabolism and nitrate environments are the two main processes for microorganisms to obtain nutrients and energy (Condron et al., 2010). Additionally, we found that ANRA and DNRA functional pathways are significantly enriched in the park water environment. Although both pathways convert nitrate to ammonium, they play different roles in the ecosystem. ANRA often occurs in aerobic environments, where the produced amines are assimilated into amino acids (Castro-Barros et al., 2017). DNRA prefers anaerobic environments, where the produced amines are used for microbial growth and are also released extracellularly, providing a nitrogen source for other bacteria (Castro-Barros et al., 2017). The rich ANRA and DNRA gene pathways in the water environment, compared to the soil environment, indicate a greater potential for nitrate to ammonium conversion. Another glutamate metabolism process is also significantly enriched in the park water environment, converting ammonia to glutamate, which is essential for microbial growth and protein synthesis (Walker and van der Donk, 2016). We infer that due to the high nitrogen content in the park water environment, the conversion of glutamine to ammonia is weaker, inhibiting nitrogen accumulation in the water environment. Therefore, excess nitrogen in the water is used to synthesize amino acids for microbial growth. Furthermore, instead of focusing on a specific denitrification gene marker pathway, we detected all denitrification genes in the park environment through metagenomic sequencing, including *narG*, *narH*, *narI*, *napA*, *nirk*, and *nosZ*. Among these, *nirk* is much more abundant in the park soil and water environments than other denitrification genes, indicating that the conversion of nitrite to nitric oxide may be the main denitrification process in the park environment, and nitric oxide is a key pollutant in urban environmental pollution (Dunlea et al., 2007). Unlike natural river systems (Wang et al., 2022), we did not detect the *norB* genes involved in the conversion of nitric oxide (NO) to nitrous oxide (N2O). This may be due to the low abundance of *norB* genes in the park environment, making them undetectable. Care should be taken when interpreting these low-abundance genes with metagenomic methods, and PCR detection or gene chips may compensate for this issue.

### 4.3 Different factors affect nitrogen cycle genes in park soil and water

Our results show that the factors affecting nitrogen cycle genes in soil and water are different. One possible reason is the different nutrient conditions in soil and water, such as higher total phosphorus in soil and higher carbon, nitrogen, and phosphorus in the water environment, which affect the composition and structure of nitrogen cycle microorganisms. Another reason is the different microbial communities in soil and water, leading to significant differences in nitrogen cycle microorganisms and thus nitrogen cycle genes. Through random forest analysis, we found that total sulfur and salinity are key predictors of soil nitrogen cycle genes, which differs from previous studies that considered phosphorus, carbon, and nitrogen as important predictors of nitrogen cycle genes (Hu et al., 2022). Bacterial community and geographical location are important predictors for the park water environment, which differs from river ecosystem studies that considered the carbon-to-nitrogen ratio as the most important factor affecting water environment nitrogen cycle genes (Wang et al., 2022). The factors affecting nitrogen cycle in urban park environments have unique characteristics compared to other ecosystems, possibly related to environmental physicochemical characteristics and human activities, requiring further research.

Only two genes, *narB* and *narH*, are positively correlated with total nitrogen (TN) in grassland soil, while eight genes such as *narH*, *nirA*, *nirK*, *GDH2*, *glnA*, *hcp*, *ureC*, and *gltB* are positively correlated with water environment TN, with other genes showing no significant correlation with environmental TN. The *nasB* gene encodes nitrite reductase (Ogawa et al., 1995) that catalyzes the critical reduction of nitrite to ammonium in the assimilatory nitrate reduction pathway, enabling cellular acquisition of bioavailable nitrogen. The significant positive correlation between the *nasB* gene and nitrogen content indicates an active nitrogen cycling process. Concurrently, it suggests that in nitrogen-rich environments, microorganisms may upregulate *nasB* expression to enhance assimilation, thereby supporting their growth and reproduction by utilizing the abundant nitrogen source. The *narH* gene, which significantly correlated with nitrogen content in both park grassland soil and water environments, is involved in multiple nitrogen cycle metabolic pathways (Takai, 2019), indicating its important role in the nitrogen cycle. Specifically, *narH* encodes an essential electron transfer subunit of the membrane-bound nitrate reductase (NarGHI) (Bertero et al., 2003). This enzyme complex, with its catalytic subunit encoded by *narG*, catalyzes the reduction of nitrate (NO_3_^–^) to nitrite (NO_2_^–^), which serves as the initial step in the denitrification pathway. As the gateway to the gaseous loss of nitrogen from the ecosystem, the activity of this enzyme directly influences the fate of nitrogen, determining whether it is retained in the system as nitrate or lost to the atmosphere as N_2_O or N_2_. Therefore, the significant correlations between *narH* and *narB* abundance and nitrogen content imply that processes of nitrate reduction, catalyzed by enzymes encoded by these genes, play a crucial role in regulating nitrogen storage and turnover in these park environments.

Through the PLS-PM model, we found that environmental factors affect multiple soil or water nitrogen cycle pathways, such as denitrification metabolic pathways, indicating the importance of environmental factors in the nitrogen cycle process. In grassland soil, heavy metals mainly affect the DNRA process, possibly related to electron transport involving heavy metals (Wang and Qiao, 2023). Notably, there are more factors affecting the randomness of water environment nitrogen cycle genes, while fewer in soil. We speculate that the composition of water environment nitrogen cycle genes is regulated by more environmental factors, while soil may be regulated by other unmeasured factors. Further research is needed to prove our hypothesis.

### 4.4 Metagenomic binning reveals the synergy, complementarity, and functional redundancy of nitrogen cycle genes

Through metagenomic binning, we found that the main nitrogen-fixing microorganisms in grassland soil include *Actinobacteria*, *Proteobacteria*, and *Euryarchaeota*, while in the water environment, they are mainly *Proteobacteria*, *Bacteroidetes*, and *Actinobacteria*. Previous studies have also found that *Euryarchaeota* is one of the main nitrogen cycle archaea in mangroves (Meng et al., 2022), indicating that archaea also participate in environmental nitrogen cycle processes. Interestingly, we found multiple nitrogen cycle genes linearly arranged adjacent to each other, such as *narG*-*narH*-*narJ*-*narI* or *narB*-*nasA*-*nirA*. The former mainly participates in the denitrification process, while the latter mainly participates in the assimilatory nitrate reduction pathway. This indicates that some nitrogen cycle genes are arranged adjacently on gene islands, synergistically participating in some nitrogen cycle pathways, possibly improving nitrogen conversion efficiency. In many other bins, the *napA* gene, which participates in the reduction of nitrate to nitrite, often co-occurs with *narG* or *nrfC* (a gene involved in energy metabolism), indicating that multiple microorganisms can participate in the same nitrogen cycle pathway, proving the functional redundancy and complementarity of microorganisms. These results indicate that the completion of certain nitrogen cycle pathways relies on multiple related genes arranged adjacently to synergistically participate, demonstrating the synergy, complementarity, and functional redundancy of environmental nitrogen cycle pathways.

The high efficiency and stability of the nitrogen cycle are fundamentally underpinned by the intricate interplay of synergy, functional redundancy, and complementarity within microbial communities. Synergy is exemplified by the tight coupling of distinct functional genes or taxa, such as the sequential reactions of ammonia- and nitrite-oxidizing bacteria in nitrification, and the coordinated action of *nirK*/*nirS* and *nosZ* genes in denitrification, which collectively drive the targeted flux of nitrogen (Xiong et al., 2024). In parallel, functional redundancy serves as a critical buffer for ecosystem resilience, ensuring the continuity of essential processes under environmental stress or the suppression of specific taxa, by leveraging multiple functionally analogous groups. For example, both *nirK* and *nirS* genes encode nitrite reductase, their relative importance in nitrite reduction is environmentally dependent (Li and Cupples, 2021). Complementarity, moreover, weaves disparate nitrogen transformation steps into a cohesive, dynamic equilibrium. This is illustrated by the substrate-product linkage between nitrogen fixation, nitrification, and denitrification, which creates an efficient biogeochemical pipeline. Additionally, the complementarity of specialized pathways like heterotrophic nitrification-aerobic denitrification confers remarkable metabolic versatility, enabling communities to thrive in heterogeneous environments (Li and Cupples, 2021). These three mechanisms do not operate in isolation; rather, they are deeply intertwined, forging a nitrogen cycle network that is simultaneously robust and highly adaptable. Our findings of the genomic clustering and co-occurrence of nitrogen-cycling genes offer a compelling molecular-level testament to these ecological principles. This genetic architecture provides a fundamental explanation for the observed robustness and functional diversity of nitrogen cycling in the environment.

## 5 Conclusion

Through metagenomic sequencing and binning, this paper explores the diversity, assembly, and functional synergy of nitrogen cycle microorganisms and genes in urban park grassland soil and water. We found that glutamate metabolism and assimilatory nitrate reduction are the main nitrogen cycle pathways in urban park grassland soil and water. The assembly of nitrogen cycle in urban park soil and water is mainly driven by stochastic processes. Nutrient factors (such as total sulfur) are the most important factors affecting nitrogen cycle genes in park grassland soil, while the bacterial community is the most important factor determining the water environment, indicating differences in the driving factors of nitrogen cycle genes in soil and water. *narH* is a common gene marking nitrogen content in urban park soil and water, involved in multiple nitrogen cycle pathways, indicating its important role in the nitrogen cycle. Through metagenomic binning, we found multiple nitrogen cycle genes linearly arranged adjacent to each other, demonstrating the functional synergy, redundancy, and complementarity of nitrogen cycle genes. Our research has significant implications for the biochemical cycle and nitrogen pollution management in urban parks.

## Data Availability

The datasets presented in this study can be found in online repositories. The names of the repository/repositories and accession number(s) can be found in the article/Supplementary material.

## Footnotes

1
https://github.com/qichao1984/NCyc

2
https://github.com/lh3/seqtk

3
https://www.kegg.jp/
