# Supplementary material for: The hidden nitrogen nexus: stochastic assembly and linear gene synergies drive urban park microbial networks
Source: Front Microbiol. 2025 Oct 1;16:1652652. doi: 10.3389/fmicb.2025.1652652 (PMC12521161; doi:10.3389/fmicb.2025.1652652)
Supplement: Supplementary file 1 [file Data_Sheet_1.docx]

**Supplemental files**

**The Hidden Nitrogen Nexus: Stochastic Assembly and Linear Gene Synergies Drive Urban Park Microbial Networks**

Maoping Li^1#^*, Jie Bi^2#^, Xiaochen Wang^2^, Huan Li^2^

1 State Key Laboratory of Herbage Improvement and Grassland Agro-Ecosystems, and College of Pastoral Agriculture Science and Technology, Lanzhou University,

Lanzhou, Gansu 730000, China

2 Institute of Mcirobiome Frontiers and One health, School of Public Health, Lanzhou University, Lanzhou 730000, China.

#These authors contribute to this paper equally.

*Corresponding author: Maoping Li, E-mail: limp@lzu.edu.cn

**Running title**: Nitrogen cycling genes and park environments

**Supplemental files**

**Figure S1-S8**

**Table S1-S5**

**
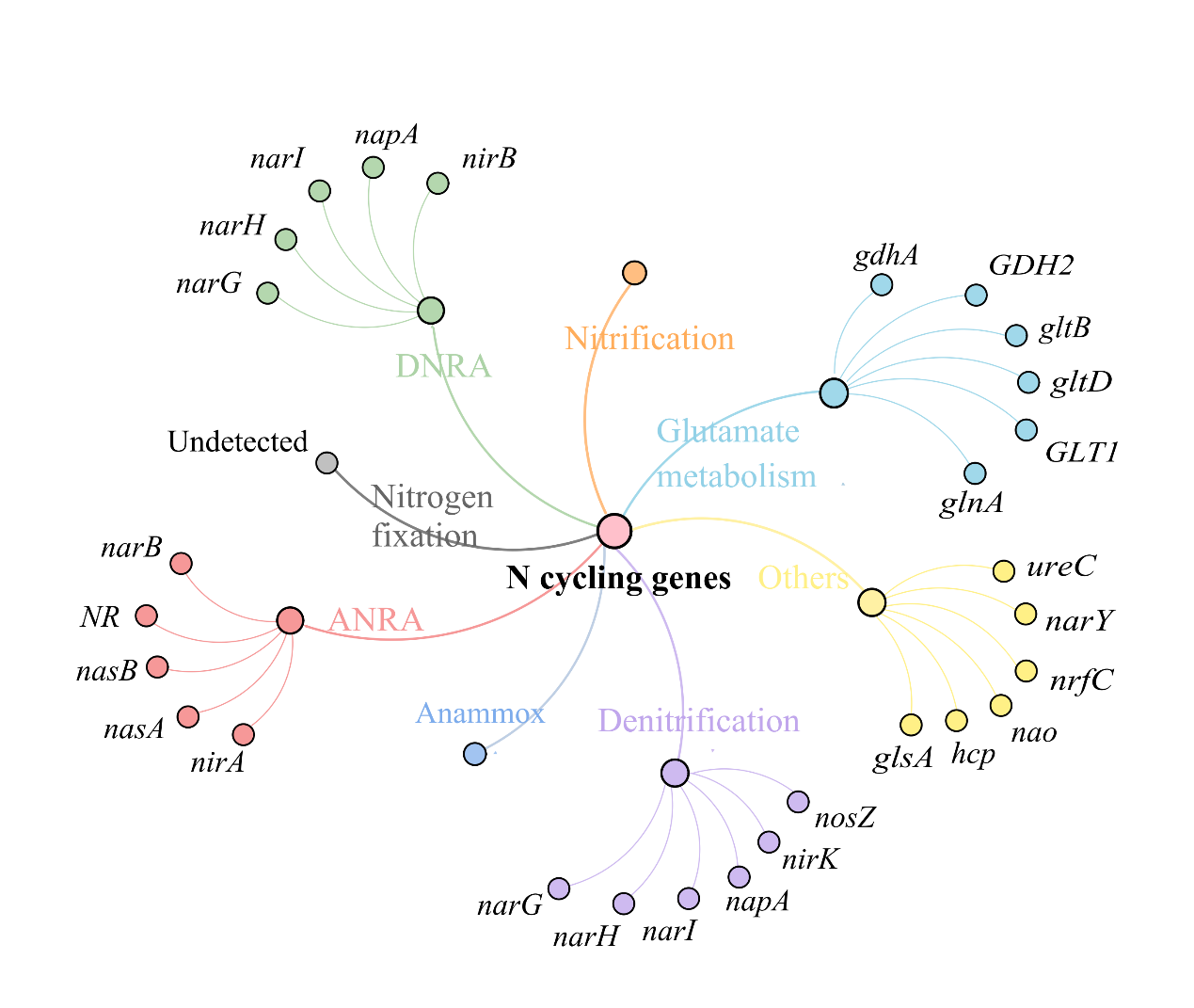
**

**Fig S1 Nitrogen cycling genes and corresponding functional pathways detected in this study**


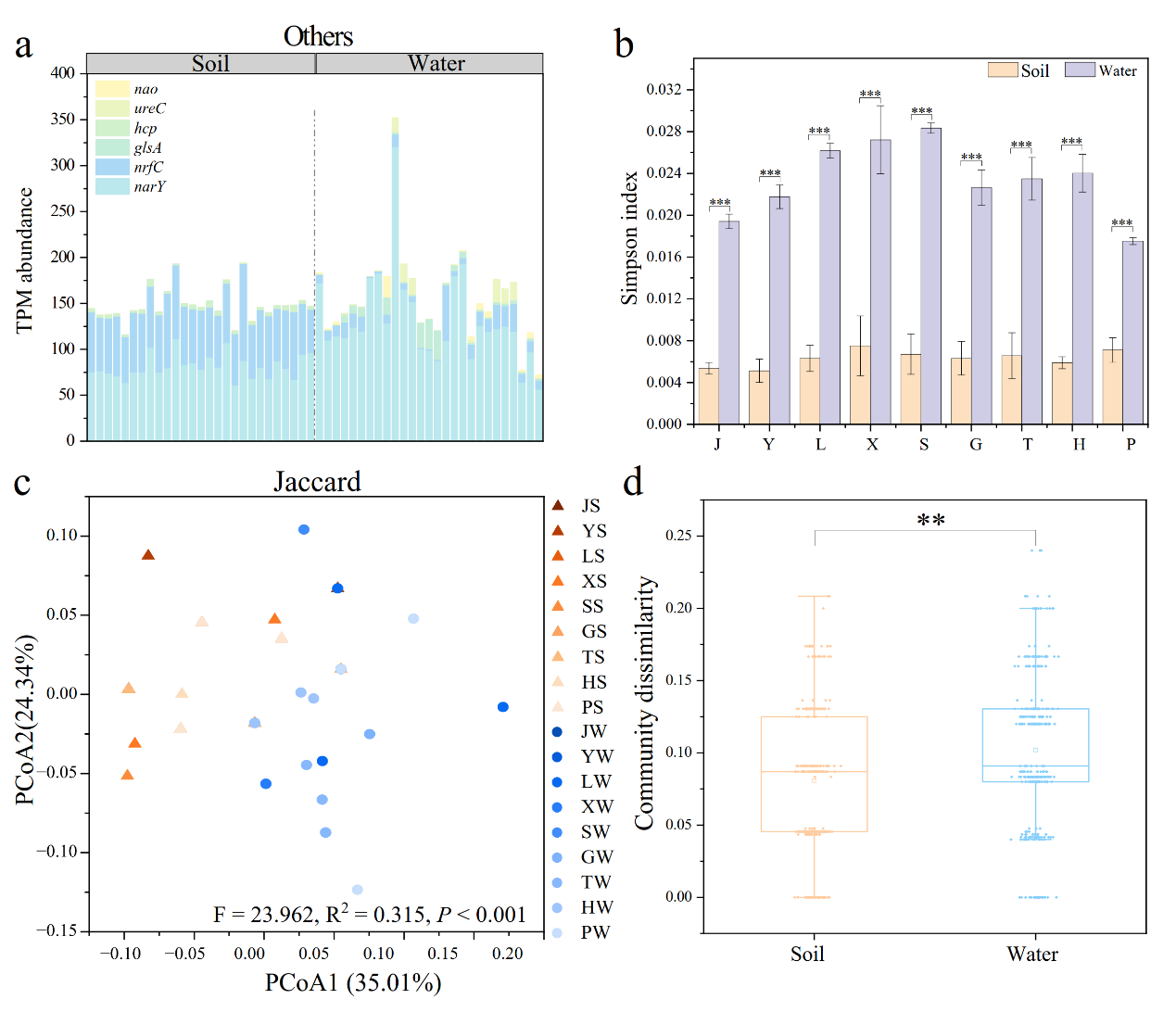


**Fig S2 TPM abundance of other categories nitrogen cycling genes (a), simpson diversity (b), beta diversity based on Jaccard distance matrix (c) and community dissimilarity (d) of nitrogen cycling genes in grassland soils and water**

**
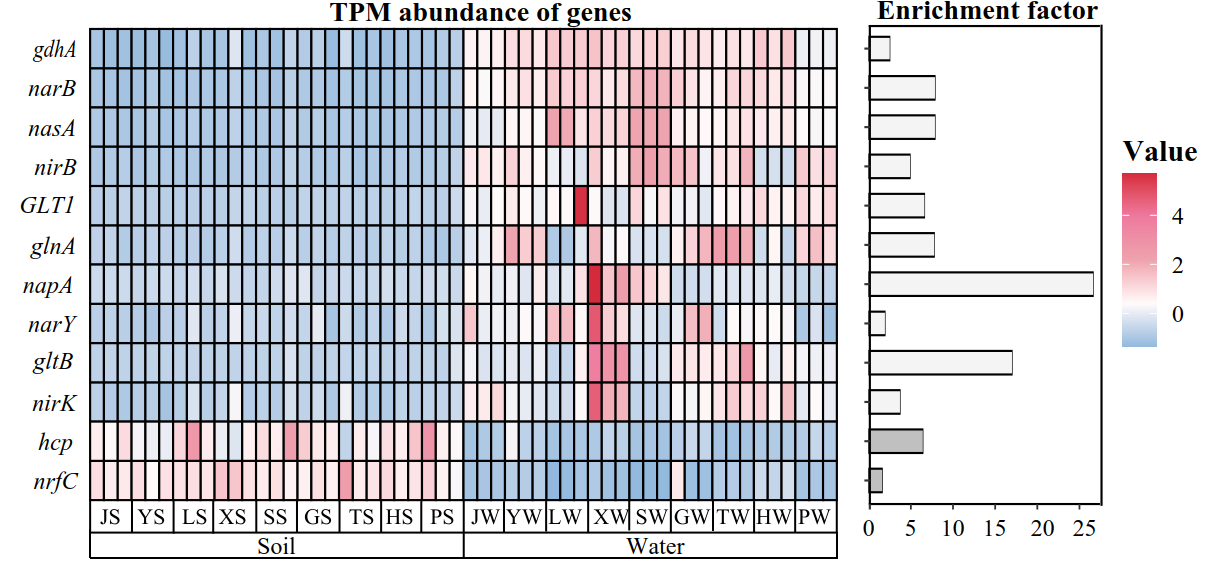
**

**Fig S3 Heatmap-enrichment histogram showed nitrogen cycling genes significantly enriched in grassland soils and water and the multiplicity of enrichment**

**
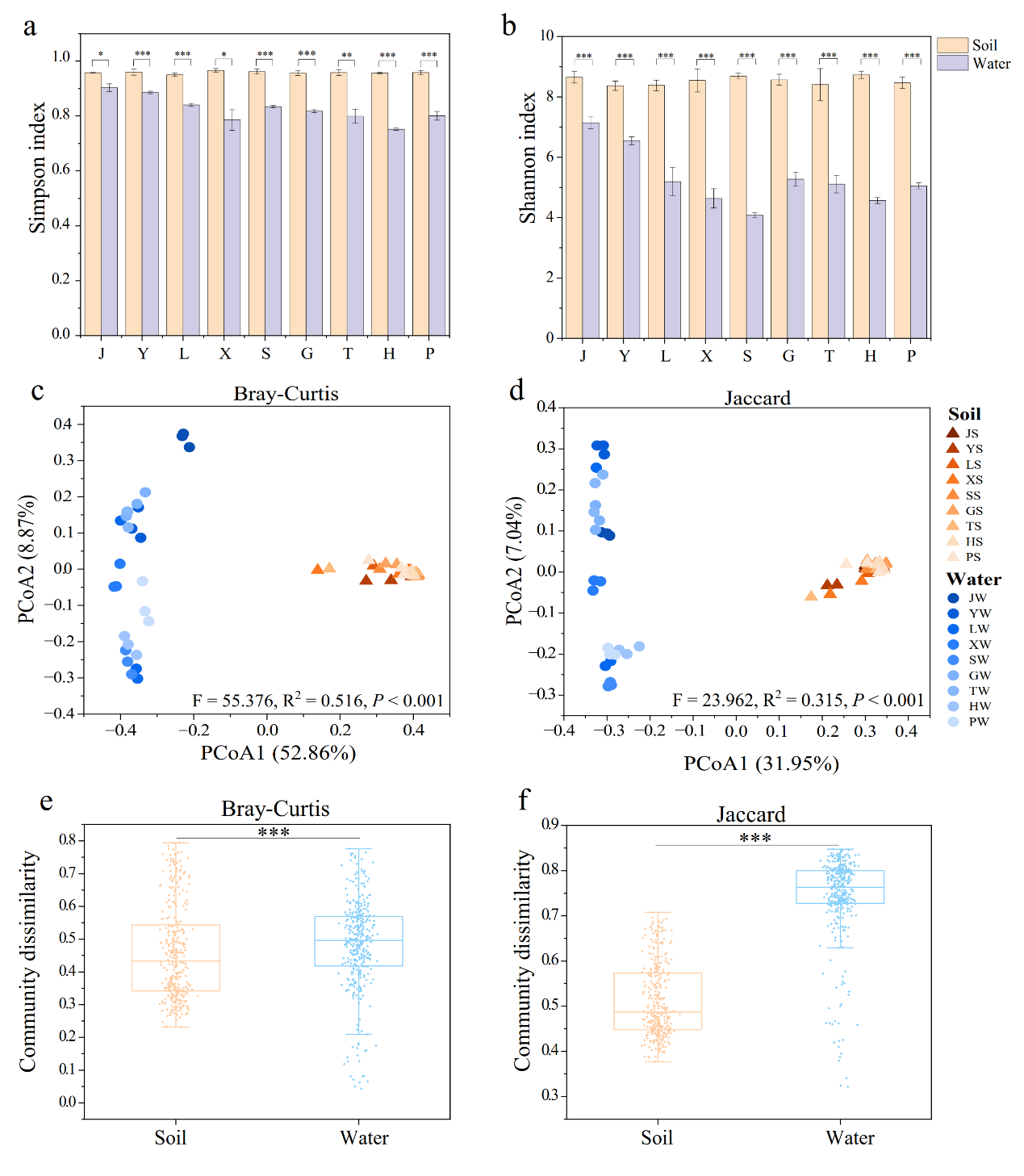
**

**Fig S4 alpha diversity (a, b), beta diversity (c, d) and community disssimilarity (e, f) of nitrogen cycling microorganisms in grassland soil and water**

**
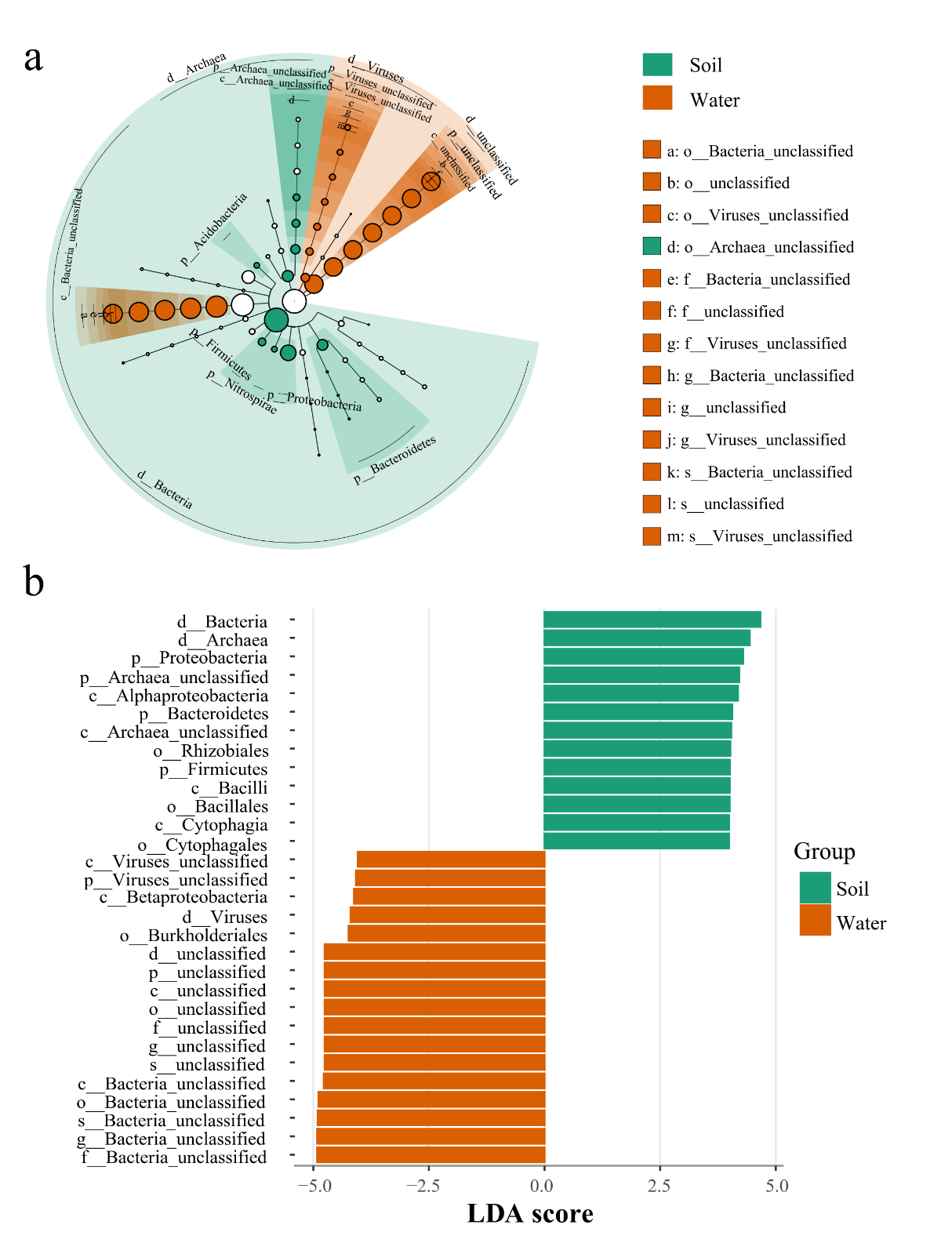
**

**Fig S5 Taxonomic hierarchical tree of nitrogen-cycling microbial species enriched in grassland soil and water analyzed by LEFSE (a). Different circles indicated different taxonomic levels, from outside to inside, in the order of kingdom-phylum-order-order-family-genera-species. Each node represented a species, and larger nodes indicated higher abundance of the species. Green color represented nitrogen cycle microorganisms that were significantly enriched in grassland soil, and orange color represents microorganisms that are significantly enriched in water. Distribution histogram (b) demonstrated significantly different species with |LDAscore| > 3.**

**
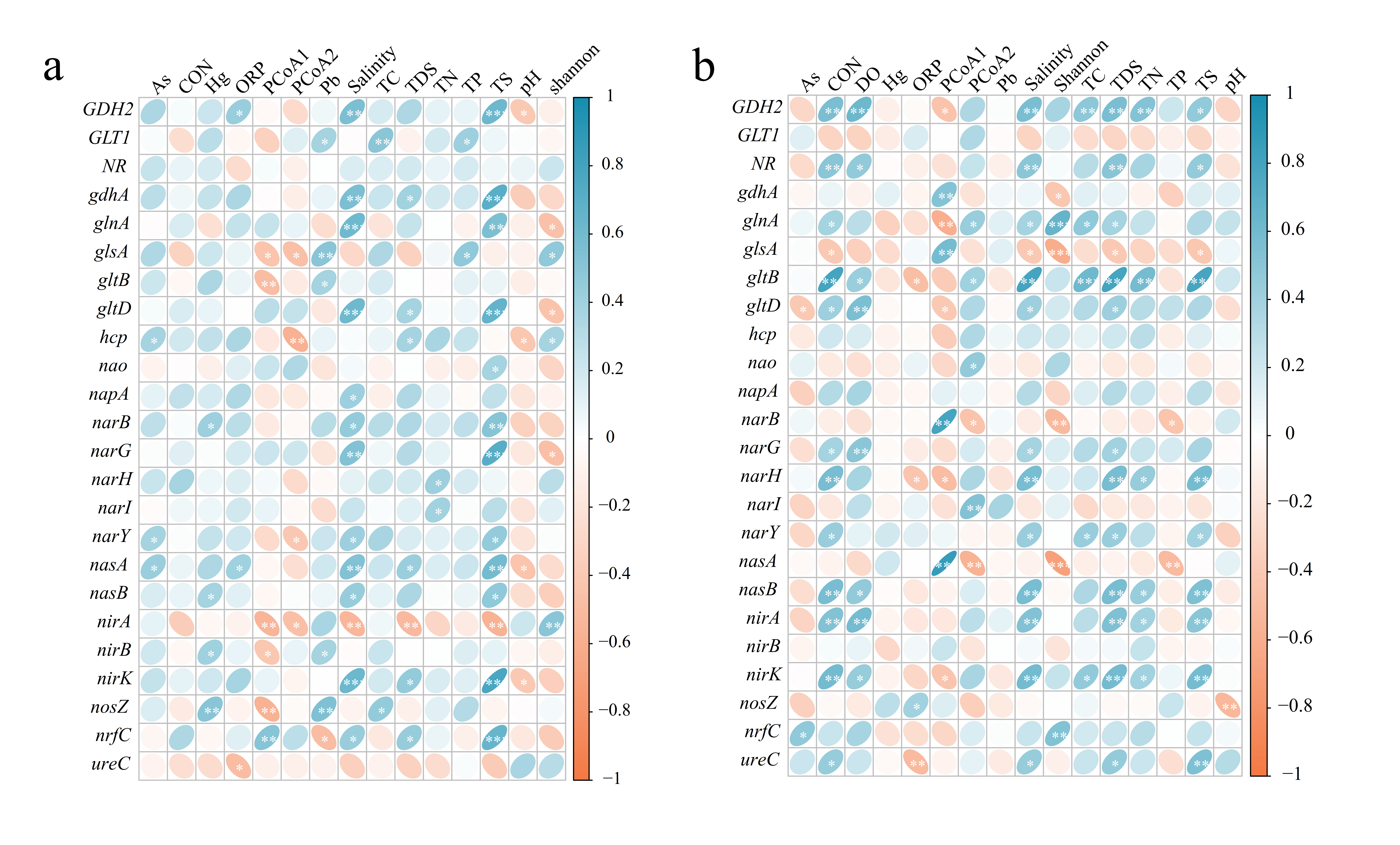
**

**Fig S6 Heatmap of Spearman’s correlation of nitrogen cycling genes with environmental physicochemical factors and bacterial community diversity in park grassland soil (a) and water (b).**

**
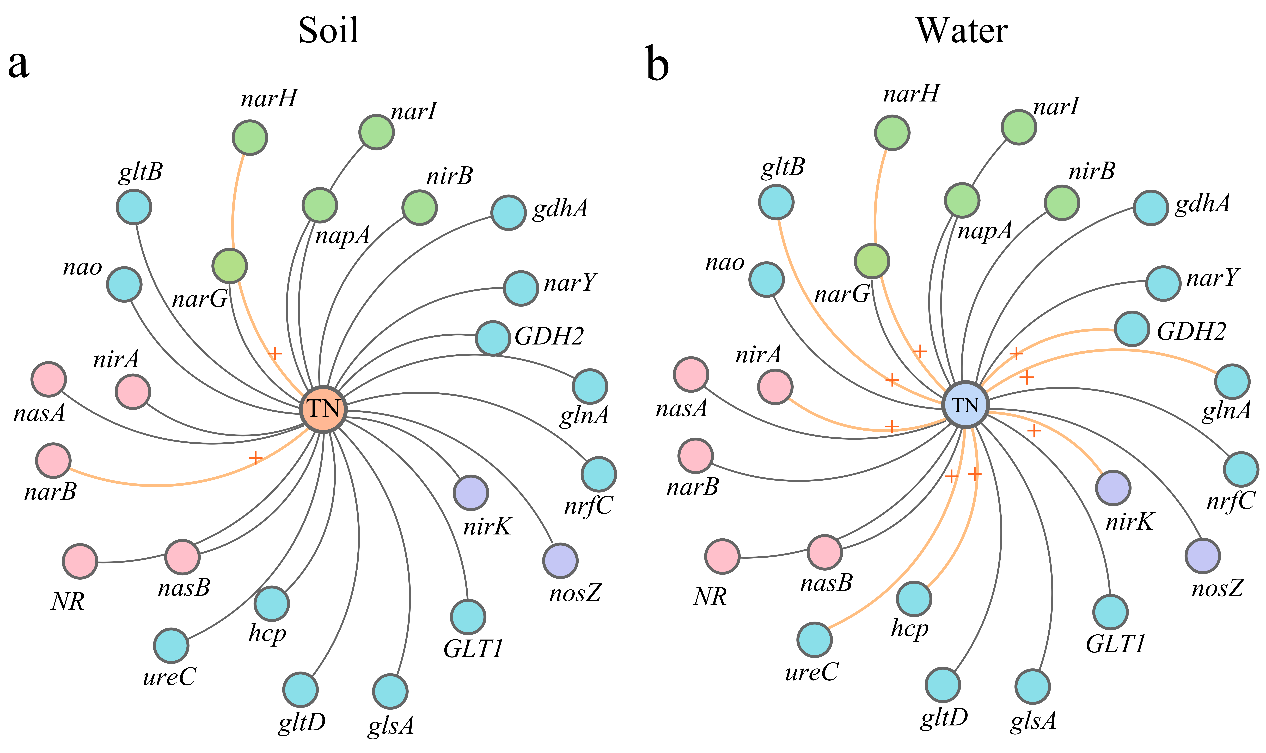
**

**Fig S7 Correlation of TN with nitrogen cycling genes in park grassland soil (a) and water (b). Orange lines represented genes significantly correlated with TN, and gray lines represented non-significant correlations. Different colored circles represent genes of different functional pathways.**

**
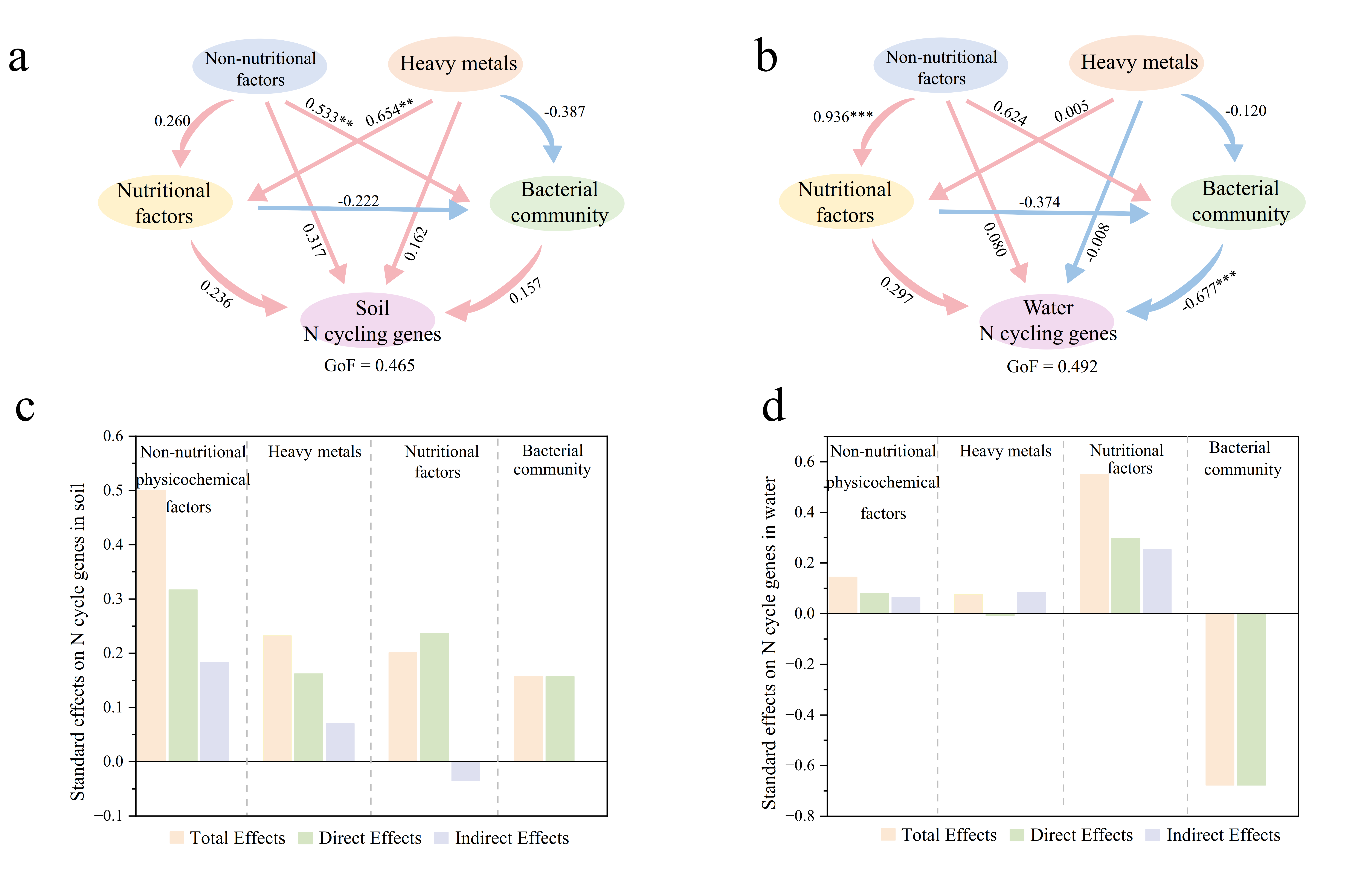
Fig S8 Potential mechanisms of influence on nitrogen cycling genes in park grassland soils and water based on partial least squares pathway modeling. Direct and indirect effects of nutrient factors, non-nutrient physicochemical factors, heavy metals, and bacterial community diversity on nitrogen cycling genes in grassland soils (a) and water (b) and standardized effects of each predictor (c, d). The pink and blue arrows in the figure indicate positive and negative effects, respectively, and the numbers next to the arrows represent standardized path coefficients. * indicated *P* < 0.05, ** indicated *P* < 0.01, and *** indicated *P* < 0.001. Abbreviation: GoF, goodness of fit.**

**Table S1 PERMANOVA test for differences in community structure of nitrogen cycling genes in grassland soil and water**

|  | Bray-Curtis | | |  | Jaccard | | |
| --- | --- | --- | --- | --- | --- | --- | --- |
|  | F | R^2^ | *P* |  | F | R^2^ | *P* |
| Soil vs. Water | 409.410 | 0.883 | **0.001***** |  | 18.849 | 0.266 | **0.001***** |
| JS vs. JW | 674.510 | 0.994 | 0.100 |  | 6.873 | 0.632 | 0.100 |
| YS vs. YW | 296.547 | 0.987 | 0.100 |  | 5.949 | 0.598 | 0.100 |
| LS vs. LW | 347.720 | 0.989 | 0.100 |  | 3.438 | 0.462 | 0.100 |
| XS vs. XW | 43.506 | 0.916 | 0.100 |  | 3.800 | 0.487 | 0.100 |
| SS vs. SW | 150.453 | 0.974 | 0.100 |  | 3.230 | 0.447 | 0.100 |
| GS vs. GW | 151.043 | 0.974 | 0.100 |  | 4.866 | 0.549 | 0.100 |
| TS vs. TW | 87.309 | 0.956 | 0.100 |  | 1.407 | 0.260 | 0.300 |
| HS vs. HW | 256.518 | 0.985 | 0.100 |  | 0.889 | 0.182 | 0.600 |
| PS vs. PW | 229.858 | 0.983 | 0.100 |  | 3.806 | 0.488 | 0.100 |

**Table S2 Significance test for ANOVA analysis of differences in relative abundance of nitrogen cycling genes in grassland soil and water**

| Genes | F | *P* |
| --- | --- | --- |
| *narB* | 458.113 | **<0.001***** |
| *gdhA* | 436.744 | **<0.001***** |
| *nasA* | 179.100 | **<0.001***** |
| *nrfC* | 257.245 | **<0.001***** |
| *nirB* | 134.497 | **<0.001***** |
| *hcp* | 99.816 | **<0.001***** |
| *glnA* | 53.061 | **<0.001***** |
| *GLT1* | 44.620 | **<0.001***** |
| *nirK* | 33.440 | **<0.001***** |
| *gltB* | 31.347 | **<0.001***** |
| *narY* | 24.887 | **<0.001***** |
| *napA* | 12.083 | **0.001***** |
| *glsA* | 11.049 | **0.002**** |
| *ureC* | 8.431 | **0.005**** |
| *nosZ* | 8.276 | **0.006**** |
| *narI* | 5.983 | **0.018*** |
| *nao* | 5.628 | **0.021*** |
| *GDH2* | 5.415 | **0.024*** |
| *nirA* | 4.877 | **0.032*** |
| *nasB* | 3.154 | 0.082 |
| *narG* | 1.830 | 0.182 |
| *NR* | 0.658 | 0.421 |
| *gltD* | 0.283 | 0.597 |
| *narH* | 0.147 | 0.703 |

**Table S3 PERMANOVA test for differences in the structure of nitrogen cycling microbial communities in grassland soils and water**

|  | Bray-Curtis | | |  | Jaccard | | |
| --- | --- | --- | --- | --- | --- | --- | --- |
|  | F | R^2^ | *P* |  | F | R^2^ | *P* |
| Soil vs. Water | 55.376 | 0.516 | **0.001***** |  | 23.962 | 0.315 | **0.001***** |
| JS vs. JW | 25.984 | 0.867 | 0.100 |  | 252.540 | 0.984 | 0.100 |
| YS vs. YW | 12.392 | 0.756 | 0.100 |  | 76.786 | 0.950 | 0.100 |
| LS vs. LW | 73.845 | 0.949 | 0.100 |  | 50.098 | 0.926 | 0.100 |
| XS vs. XW | 49.654 | 0.925 | 0.100 |  | 51.634 | 0.928 | 0.100 |
| SS vs. SW | 143.293 | 0.973 | 0.100 |  | 172.432 | 0.977 | 0.100 |
| GS vs. GW | 221.882 | 0.983 | 0.100 |  | 237.205 | 0.983 | 0.100 |
| TS vs. TW | 48.860 | 0.924 | 0.100 |  | 27.955 | 0.875 | 0.300 |
| HS vs. HW | 690.242 | 0.994 | 0.100 |  | 184.229 | 0.979 | 0.600 |
| PS vs. PW | 170.574 | 0.977 | 0.100 |  | 131.570 | 0.970 | 0.100 |

**Table S4 ANOVA analysis of nitrogen cycling microorganisms enriched in grassland soil and water respectively top10**

| Genus | F | *P* |
| --- | --- | --- |
| *Planctomycetes_noname* | 160.675 | **<0.001***** |
| *Faecalibacterium* | 135.769 | **<0.001***** |
| *Phycisphaeraceae_noname* | 133.883 | **<0.001***** |
| *Gemmata* | 131.322 | **<0.001***** |
| *Pelotomaculum* | 122.944 | **<0.001***** |
| *Gemmatimonadetes_noname* | 115.108 | **<0.001***** |
| *Methylobacterium* | 114.258 | **<0.001***** |
| *Singulisphaera* | 113.145 | **<0.001***** |
| *Corallococcus* | 111.426 | **<0.001***** |
| *Methylocaldum* | 110.052 | **<0.001***** |
| *Limnohabitans* | 75.541 | **<0.001***** |
| *Polynucleobacter* | 60.511 | **<0.001***** |
| *Paucibacter* | 56.135 | **<0.001***** |
| *Viruses_unclassified* | 46.632 | **<0.001***** |
| *Comamonadaceae_noname* | 45.388 | **<0.001***** |
| *Burkholderiales_noname* | 39.559 | **<0.001***** |
| *Candidatus_Planktophila* | 38.214 | **<0.001***** |
| *Burkholderia* | 36.566 | **<0.001***** |
| *Betaproteobacteria_noname* | 34.560 | **<0.001***** |
| *Alphaproteobacteria_noname* | 33.587 | **<0.001***** |

**Table S5 Topological characterization of gene co-occurrence networks for nitrogen cycling in park grassland soils and water**

|  | Nodes | Edges | Average degree | Average path length | Graph diameter | Graph density | Clustering coefficient | Modularity | Positive (%) |
| --- | --- | --- | --- | --- | --- | --- | --- | --- | --- |
| Soil | 24 | 62 | 5.167 | 2.111 | 5 | 0.225 | 0.727 | 0.118 | 90.323 |
| Water | 24 | 80 | 6.667 | 1.993 | 4 | 0.290 | 0.549 | 0.364 | 72.500 |
